# Effect of Instrumental Indian Classical Music on Blood Pressure, Body Surface Temperature, and Oxygen Saturation Among Healthy Elderly Individuals in Datia District: A Physiological Study

**DOI:** 10.7759/cureus.104042

**Published:** 2026-02-21

**Authors:** Palak Shilpi, Mahima Pareek, Rishi Rajpoot, Vivek Verma, Vishal Soni

**Affiliations:** 1 Physiology, Government Medical College, Datia, Datia, IND

**Keywords:** blood pressure, elderly, indian classical music, music therapy, oxygen saturation, surface body temperature

## Abstract

Background

The use of music therapy for modulating physiological parameters has gained interest in geriatric care, yet its specific effects in healthy elderly populations remain underexplored. This study aimed to assess the immediate impact of instrumental Indian classical music on blood pressure (BP), surface body temperature (ST), and oxygen saturation (SpO2) in older adults.

Objectives

To evaluate the immediate effects of instrumental Indian classical music on blood pressure, surface body temperature and SpO2 in older adults.

Methods

This exploratory physiological study recruited 96 elderly participants aged 60 years and above. Participants underwent a 15-minute exposure to instrumental Indian classical music. Physiological parameters, including systolic and diastolic BP, ST, and SpO2, were recorded at four time points: pre-intervention, during intervention, immediately post-intervention, and 5 minutes post-intervention. Participants rested in a standardised, quiet, temperature-controlled environment before measurements to minimise acute environmental influences. Potential short-term confounders such as recent caffeine intake, exertion, and ambient noise were controlled, while demographic variables were documented and analysed descriptively. Statistical analysis involved Repeated Measures ANOVA and the Wilcoxon Rank test.

Results

A significant reduction in both systolic and diastolic BP was observed immediately after the intervention, indicating a calming effect of music on cardiovascular parameters. However, surface body temperature showed only a minimal decrease, while SpO2 remained relatively unchanged throughout the measurements.

Conclusion

The study demonstrates that exposure to instrumental Indian classical music can effectively reduce blood pressure in healthy older adults, suggesting its potential role as a non-pharmacological intervention for cardiovascular health and stress reduction. Minimal changes in surface body temperature and SpO2 indicate that these parameters may be less responsive to short-term music interventions. Future research should investigate the long-term benefits and broader applicability of music therapy in elderly populations with varied health conditions.

## Introduction

The influence of music on physiological parameters such as blood pressure, heart rate, and overall cardiovascular health has garnered increasing attention as a non-pharmacological health intervention. As the global population ages rapidly, understanding how various music genres impact physiological functions in older adults is crucial for developing effective, low-risk therapeutic strategies. Although music has long been associated with healing and relaxation, empirical evidence regarding its physiological effects, particularly in elderly populations, remains inconsistent and limited. Research indicates that different types of music can significantly impact cardiovascular health. For example, classical music has been shown to lower systolic blood pressure (SBP) and heart rate, thereby inducing relaxation [1], while also improving mood, reducing tension, and enhancing mental clarity, which indirectly supports cardiovascular well-being [2]. Music exerts its effects primarily by stimulating the autonomic nervous system, influencing heart rate variability and promoting relaxation and stress reduction [3].

Pleasurable music further modulates emotional responses through dopamine release in the brain [4,5]. Certain forms of music, including flute-based compositions, have been associated with improved sleep quality, an important determinant of overall health in older adults [6], and music interventions have demonstrated efficacy in reducing preoperative anxiety, highlighting their therapeutic potential [7]. Despite these findings, evidence on the effects of Indian classical music, particularly in community-dwelling elderly individuals, remains sparse, underscoring the need for further research. Raag Malkauns is one of the oldest and most revered ragas in Indian classical music, traditionally associated with serenity and introspection [8]. Belonging to the Bhairavi thaat, it follows a pentatonic scale dominated by komal swaras, producing a meditative and calming tonal quality. Typically performed during evening and late-night hours, the raga is believed to facilitate mental relaxation and parasympathetic predominance. Its slow tempo and minimalistic melodic structure make it especially suitable for therapeutic applications. Instrumental, flute-based renditions enhance these effects by providing continuous, breath-like sound patterns without lyrical distraction.

Ageing is associated with progressive autonomic imbalance characterized by reduced parasympathetic tone and relative sympathetic predominance, contributing to elevated blood pressure and cardiovascular vulnerability. Although music-based interventions have demonstrated potential for autonomic modulation, most available evidence derives from clinical or hospital-based populations. There remains limited physiological evidence in community-dwelling healthy elderly individuals, particularly in the context of Indian classical music.

Raag Malkauns, a pentatonic raga traditionally associated with introspection and meditative calm, is characterized by slow tempo and sustained tonal progression. Such structural features may influence autonomic balance through modulation of emotional and neural pathways. However, empirical physiological evaluation of this raga in elderly individuals remains sparse.

Therefore, this study aimed to examine acute within-subject changes in blood pressure, surface temperature (ST), and oxygen saturation following exposure to standardized instrumental Raag Malkauns in healthy elderly participants.

## Materials and methods

Study design

A community-based repeated-measures physiological study with each participant serving as their own control was conducted. Baseline rest measurements functioned as the internal control condition to reduce inter-individual variability.

Study setting

The study was conducted in and around Government Medical College (GMC), Datia, Madhya Pradesh, within the Department of Physiology. All physiological recordings were performed in a designated soundproof room maintained at ambient temperature (24-26 °C) to minimize environmental variability.

Sampling technique and participants

Participants were recruited using convenience sampling from community centers, retirement homes, and social clubs located in the study area. Individuals aged 60 years and above were approached and screened for eligibility.

Sample size

A total of 100 elderly individuals were initially recruited. One participant declined consent, and three participants were excluded due to conductive hearing loss detected on auditory screening using Rinne’s test. Thus, the final effective sample size was 96 participants.

Inclusion criteria

Adults aged 60 years or above who were non-smokers, non-alcohol consumers, and free from significant auditory or cognitive impairment that could interfere with music perception were included in the study. Participants were required to provide written informed consent.

Exclusion criteria

Individuals with a history of auditory or cognitive impairment affecting music perception, diagnosed cardiovascular disorders, chronic pain conditions, or any contraindication to music exposure were excluded from the study, as were those unwilling to participate.

Intervention and data collection procedure

After obtaining written informed consent, auditory screening was conducted using Rinne’s and Weber’s tuning fork tests [9,10]. Participants were instructed to refrain from caffeine intake and vigorous physical activity for at least two hours prior to assessment. Each participant was positioned supine on an examination couch and allowed to rest for five minutes to achieve physiological stabilization. Demographic variables including age, sex, education, residence, and comorbidity status were recorded using a semi-structured questionnaire on interview basis.

Baseline measurements of SBP and diastolic blood pressure (DBP) were recorded using a calibrated digital sphygmomanometer, body surface temperature was measured using a non-contact infrared thermometer at a standardized anatomical site, and peripheral oxygen saturation (SpO₂) was recorded using a fingertip pulse oximeter. All measurements were taken on the same side of the body for consistency.

A separate external control group (e.g., alternate music or silent rest with headphones) was not included due to logistical constraints and the exploratory nature of this pilot physiological study. To partially compensate, participants served as their own controls through pre-intervention baseline measurements taken after a standardized five-minute rest in a soundproof environment.

Participants were then exposed to a standardized 15-minute session of flute-based instrumental Indian classical music (Raag Malkauns) delivered through external noise-cancelling headphones at a comfortable listening level (50-60 dB). Participants were instructed to remain relaxed, motionless, and awake during the session. Physiological parameters were recorded once during the intervention, immediately at the end of the music session, and additional measurements of body surface temperature and SpO₂ were obtained five minutes post-intervention.

All devices were calibrated according to manufacturer guidelines prior to data collection. Blood pressure was measured using a validated automated digital sphygmomanometer with the cuff placed on the non-dominant arm at heart level. ST was measured at the mid-forehead region using a non-contact infrared thermometer. SpO₂ was recorded using a standardized fingertip pulse oximeter applied to the index finger of the same hand for all measurements.

All measurements were obtained by the same trained assessor to minimize inter-observer variability. No missing data were observed in the final dataset.

Control of potential confounders

Participants were instructed to avoid caffeine, heavy meals, smoking, alcohol, and vigorous activity for at least two hours prior to assessment to minimize short-term physiological variability. Measurements were performed at similar times of day in a temperature-controlled room (24-26 °C). Individuals with known cardiovascular disease, acute illness, or medications affecting blood pressure were excluded. Although laboratory parameters (e.g., glucose, lipid profile), diet quality, psychosocial stress, and long-term lifestyle factors were not formally measured, the homogeneous healthy cohort and within-subject comparison design were intended to reduce confounding. These unmeasured factors may introduce residual bias and limit causal inference.

Statistical analysis

Data were analyzed using appropriate statistical software. Continuous variables were expressed as mean ± standard deviation. Normality was assessed using the Shapiro-Wilk test. Systolic and diastolic blood pressure did not meet normality assumptions (p < 0.05), and therefore non-parametric tests were applied. Within-subject comparisons between baseline and post-intervention measurements were performed using the Wilcoxon signed-rank test. Time-related differences across multiple measurements were assessed using the Friedman test, followed by Durbin-Conover post-hoc analysis with Bonferroni adjustment. Correlation analysis was conducted exploratorily to evaluate coordinated physiological changes between parameters. A p-value < 0.05 was considered statistically significant.

Ethical considerations

The study was conducted in accordance with the principles of the Declaration of Helsinki. Ethical approval was obtained from the Institutional Ethics Committee of Government Medical College, Datia, prior to initiation of the study (approval 67/Physio/GMC/IECBMHR/2024). Written informed consent was obtained from all participants after explaining the study objectives, procedures, and voluntary nature of participation. Participant confidentiality and anonymity were strictly maintained, and data were used solely for research purposes. Participants were informed of their right to withdraw at any time without penalty. The intervention posed minimal risk, as music exposure was non-invasive and delivered at a safe and comfortable auditory level.

## Results

A total of 96 elderly participants were included with a mean age of 68.86 ± 6.28 years. Most participants were aged ≥70 years (44.8%), followed by 60-64 years (30.2%) and 65-69 years (25.0%). Males constituted 63.5% of the cohort. Slightly more than half of the participants were from rural areas (56.2%) (Table 1).

**Table 1:** Baseline demographic characteristics of the study participants (n = 96) Values are presented as mean ± standard deviation (SD) for continuous variables and number (percentage) for categorical variables. Percentages were calculated using the total study population (n = 96) as the denominator.

Variable	Category	n (%) or Mean ± SD
Age (years)	Mean ± SD	68.86 ± 6.28
	60–64	29 (30.2%)
	65–69	24 (25.0%)
	≥70	43 (44.8%)
Sex	Male	61 (63.5%)
	Female	35 (36.5%)
Residence	Urban	42 (43.8%)
	Rural	54 (56.2%)

Gender-stratified analysis demonstrated a consistent reduction in blood pressure across both male and female participants following exposure to instrumental Indian classical music. Among males (n = 61), mean SBP declined progressively from 124 ± 16.7 mmHg at baseline to 121 ± 15.9 mmHg at five minutes post-intervention, while DBP decreased from 77.7 ± 10.4 mmHg to 74.7 ± 9.73 mmHg. Similarly, females (n = 35) showed a reduction in SBP from 126 ± 25.9 mmHg to 122 ± 23.7 mmHg and DBP from 78.0 ± 14.9 mmHg to 74.5 ± 12.7 mmHg (Table 2).

**Table 2:** Gender-wise Changes in Physiological Parameters (Mean ± SD) Values are presented as mean ± standard deviation (SD). Physiological parameters including systolic blood pressure (SBP), diastolic blood pressure (DBP), surface temperature (ST), and peripheral oxygen saturation (SpO₂) were recorded at four time points: pre-intervention (baseline), during music exposure, immediately post-intervention, and five minutes post-intervention. Gender-wise subgroup analysis was performed to evaluate temporal changes following exposure to instrumental Indian classical music. SBP and DBP are expressed in millimetres of mercury (mmHg), surface temperature in degrees Fahrenheit (°F), and SpO₂ as percentage saturation. n indicates the number of participants in each subgroup.

Parameter	Time point	Male (n=61)	Female (n=35)
SBP (mmHg)	Pre	124 ± 16.7	126 ± 25.9
	During	123 ± 16.1	125 ± 24.8
	Post	122 ± 16.3	124 ± 24.0
	5 min after	121 ± 15.9	122 ± 23.7
DBP (mmHg)	Pre	77.7 ± 10.4	78.0 ± 14.9
	During	76.3 ± 9.99	76.9 ± 13.9
	Post	75.6 ± 9.95	76.3 ± 13.4
	5 min after	74.7 ± 9.73	74.5 ± 12.7
Surface Temp (°F)	Pre	97.5 ± 0.62	97.8 ± 0.73
	During	97.6 ± 0.55	97.7 ± 0.65
	Post	97.6 ± 0.54	97.8 ± 0.68
	5 min after	97.5 ± 0.58	97.7 ± 0.75
SpO₂ (%)	Pre	97.6 ± 0.91	97.8 ± 0.91
	During	97.7 ± 0.95	97.9 ± 1.00
	Post	97.9 ± 0.81	97.9 ± 0.94
	5 min after	97.8 ± 0.87	98.3 ± 0.70

The Shapiro-Wilk test showed non-normal distributions for SBP (p < 0.001) and DBP (p < 0.05), prompting the use of non-parametric tests. The Wilcoxon signed-rank test confirmed significant reductions in SBP (W = 2911, p < 0.001) and DBP (W = 2994, p < 0.001), with an average decrease of approximately 2.5 mmHg. ST also showed a small but significant reduction (W = 1045, p = 0.004), whereas SpO₂ did not show a statistically significant change (W = 739, p = 0.075) (Table 3).

**Table 3:** Pre- and Post-Intervention Changes in Physiological Parameters Values represent pre–post differences. SE = standard error; CI = confidence interval; SBP = systolic blood pressure; DPB = diastolic blood pressure; SpO₂ = peripheral oxygen saturation. *Statistically significant (p < 0.05). *p<0.05, **p<0.01, ***p<0.001

Parameter	Test statistic (W)	Mean difference	SE	95% CI (Lower–Upper)	p-value
SBP (mmHg)	2911	2.5	0.5345	1.50–3.50	<0.001***
DBP (mmHg)	2994	2.5	0.3535	1.50–3.00	<0.001***
Surface Temp (°F)	1045	−0.10	0.0292	−0.15–−0.05	0.004**
SpO₂ (%)	739	−0.00005	0.1043	−1.00–0.00	0.075 NS

The Friedman test demonstrated significant time-related differences for systolic and diastolic blood pressure across measurement points. Although statistical variation was observed for SpO₂ across time, the magnitude of change remained clinically negligible and within normal physiological limits (χ² = 1071, df = 15, p < 0.001).

Pairwise comparisons revealed that SBP and DBP values were significantly higher at baseline than during the intervention, immediately after, and five minutes post-intervention (all p < 0.001), indicating a sustained blood pressure-lowering effect. The observed mean reduction of approximately 2.5 mmHg in both systolic and diastolic blood pressure represents a modest short-term physiological change. While statistically significant, its immediate clinical impact at the individual level is limited.

SpO₂ decreased significantly during the intervention and further after the intervention (both p < 0.001), although the magnitude of change remained within normal physiological limits. ST decreased significantly between baseline and during the intervention (p = 0.030) and between baseline and post-intervention (p < 0.001) (Table 4).

**Table 4:** Pairwise Comparison of SpO₂, Blood Pressure, and Surface Temperature Across Different Measurement Time Points Overall differences across time points were evaluated using the Friedman repeated-measures test (χ² = 1071, df = 15, Kendall’s W = 0.78, p < 0.001). Pairwise comparisons were performed using the Durbin-Conover post-hoc test with Bonferroni adjustment. A p-value < 0.05 was considered statistically significant. NS = not significant (p>0.05). Statistical significance was defined as *p < 0.05, **p < 0.01, and ***p < 0.001

Parameter	Pairwise comparison (Durbin–Conover)	Test statistic	p-value
Systolic blood pressure (SBP)	Pre vs During	0.24	0.811 NS
	Pre vs Post	2.068	0.039*
	Pre vs 5 min	3.717	<0.001***
	During vs Post	1.828	0.068 NS
	During vs 5 min	3.477	<0.001 ***
	Post vs 5 min	1.648	0.099 NS
Diastolic blood pressure (DBP)	Pre vs During	1.064	0.288 NS
	Pre vs Post	2.832	0.005**
	Pre vs 5 min	4.541	<0.001***
	During vs Post	1.768	0.077 NS
	During vs 5 min	3.477	<0.001***
	Post vs 5 min	1.708	0.088 NS
Surface temperature (ST)	Pre vs During	2.173	0.03 *
	Pre vs Post	1.858	0.063 NS
	During vs Post	0.315	0.753 NS
Oxygen saturation (SpO₂)	Pre vs During	0.914	0.361 NS
	Pre vs Post	1.843	0.065 NS
	Pre vs 5 min	2.563	0.01*
	During vs Post	0.929	0.353 NS
	During vs 5 min	1.648	0.099 NS
	Post vs 5 min	0.719	0.472 NS

**Figure 1:**
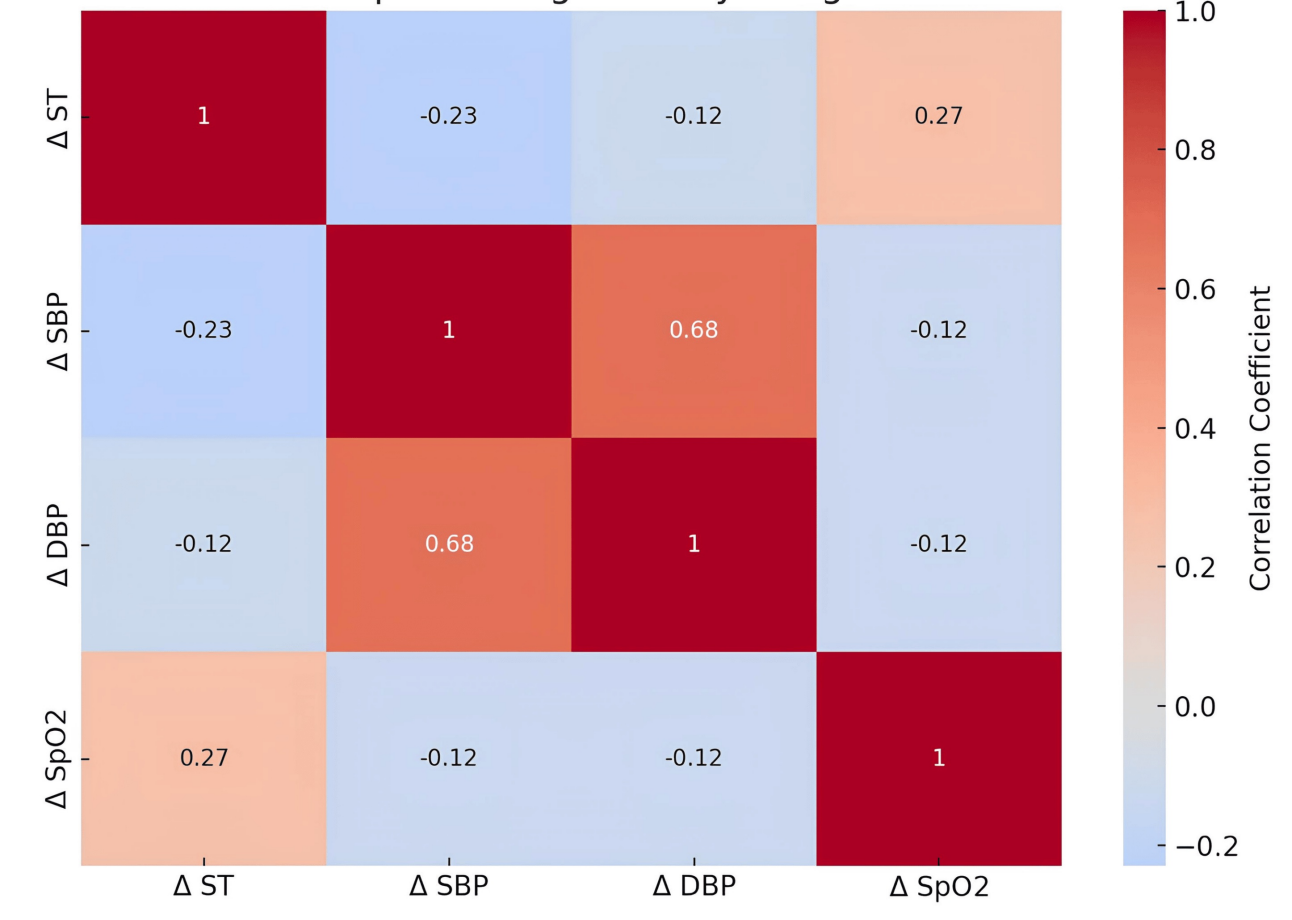
Correlation Matrix Showing the Relationship Between Changes in Physiological Parameters Following the Procedure The heatmap illustrates pairwise correlation coefficients among changes (Δ) in surface temperature (ST), systolic blood pressure (SBP), diastolic blood pressure (DBP), and peripheral oxygen saturation (SpO₂). Values within the cells represent correlation coefficients (r). Positive correlations are shown in warmer colors, while negative correlations are shown in cooler colors, with color intensity proportional to the strength of the correlation. Δ denotes the difference between pre-procedure and post-procedure measurements.

Correlation analysis showed a strong positive association between changes in SBP and DBP (r = 0.68). It also revealed a moderate positive correlation between changes in SpO₂ and ST (r = 0.27). In contrast, changes in SBP correlated negatively with changes in ST (r = −0.23), as did changes in DBP (r = −0.12), suggesting that reductions in blood pressure occurred alongside subtle peripheral thermoregulatory changes (Figure 1).

Collectively, these findings demonstrate that instrumental Indian classical music induces favorable cardiovascular modulation in healthy elderly individuals while maintaining stable oxygen saturation.

## Discussion

The present study demonstrates that short-term exposure to instrumental Indian classical music (Raag Malkauns) produces a statistically significant and physiologically meaningful reduction in systolic and diastolic blood pressure among healthy elderly individuals, while maintaining stable oxygen saturation and inducing only minimal changes in body surface temperature. These findings support the growing evidence that music-based interventions can modulate cardiovascular function through autonomic mechanisms in older adults.

Ageing is associated with progressive autonomic dysregulation, characterised by reduced parasympathetic activity and relative sympathetic dominance, which contributes to increased blood pressure and heightened cardiovascular risk [11]. Large population-based datasets on healthy ageing have consistently shown a decline in heart rate variability with advancing age, underscoring the need for interventions to restore autonomic balance in older adults [12]. In this context, the observed reductions in both systolic and diastolic blood pressure in the present study suggest that instrumental Indian classical music may modulate autonomic balance in a short-term manner, although direct autonomic indices were not measured.

The strong positive correlation between changes in systolic and diastolic blood pressure (r = 0.68) indicates a coordinated cardiovascular response. This finding aligns with prior evidence showing that music can influence the cardiac autonomic nervous system, primarily by enhancing parasympathetic modulation and reducing cardiovascular arousal [13]. Systematic reviews have reported that music exposure is associated with increased heart rate variability and vagal activity, although methodological heterogeneity has been noted across studies [11]. Despite the absence of direct heart rate variability measurements in the present study, the consistent reductions in blood pressure across multiple time points indirectly support autonomic involvement.

The present findings are also consistent with studies conducted in clinical populations, where music interventions have resulted in reductions in blood pressure, heart rate, and anxiety, indicating favourable autonomic modulation [14,15]. Similar physiological benefits have been observed in patients undergoing hemodialysis, where music exposure improved oxygen saturation and reduced cardiovascular stress without adverse effects [16]. Although these studies involved patient populations, the convergence of results supports the broader cardiovascular effects of music across different health states.

Importantly, oxygen saturation remained within normal physiological limits throughout the intervention period in the present study. Although statistical analysis revealed significant changes across time points, the magnitude of SpO₂ variation was small and clinically insignificant. This finding reinforces the safety of music-based interventions in elderly individuals and is consistent with previous reports showing that music does not compromise respiratory efficiency and may even support oxygenation under certain conditions [14,16].

The modest but significant reduction in body surface temperature observed during and after the intervention may reflect peripheral vasodilation secondary to reduced sympathetic tone. The negative correlations between changes in blood pressure and surface temperature further support this interpretation, suggesting that cardiovascular relaxation occurred alongside subtle thermoregulatory adjustments. Similar peripheral physiological responses have been reported in relaxation-based interventions, including exposure to calming environments and sensory stimuli [17].

Recent neurophysiological evidence highlights that the autonomic effects of Indian classical music depend on specific musical characteristics. Sahoo et al. demonstrated that North Indian classical music with a higher ratio of minor notes may reduce parasympathetic tone and increase stress-related responses [18]. In contrast, Raag Malkauns is traditionally characterized by a slow tempo and meditative structure, which may explain the favourable cardiovascular modulation observed in the present study. This distinction underscores the importance of raga selection and musical structure when designing therapeutic music interventions and may partly account for inconsistent findings reported in some hospital-based meta-analyses [19].

Despite the absence of a separate control arm, the repeated-measures design, environmental standardization, and restriction of acute behavioral confounders improve internal consistency. Nevertheless, unmeasured confounding and expectancy effects may influence results; thus, conclusions should be interpreted cautiously and validated through randomized controlled trials.

Overall, the present study adds to the growing body of evidence supporting instrumental Indian classical music as a safe, culturally acceptable, and non-pharmacological approach for cardiovascular modulation in elderly individuals. While the cross-sectional design limits causal inference and direct autonomic indices were not measured, the consistent and sustained reductions in blood pressure observed across time points suggest a meaningful short-term physiological effect. Future studies incorporating heart rate variability analysis, longer follow-up durations, and comparative evaluation of different ragas may further elucidate underlying mechanisms and optimize clinical application in geriatric care.

Limitations 

The absence of an external control group restricts causal inference and does not allow differentiation between music-specific and nonspecific relaxation effects. Direct autonomic measures such as heart rate or heart rate variability were not recorded, limiting mechanistic interpretation. The intervention consisted of a single short-term session, precluding conclusions regarding long-term benefit. The modest magnitude of blood pressure reduction, although statistically significant, may have limited immediate clinical relevance. Furthermore, unmeasured factors such as baseline psychological state, expectancy effects, and lifestyle variables may introduce residual confounding. These findings should therefore be interpreted as preliminary physiological associations.

## Conclusions

This study demonstrates that short-term exposure to instrumental Indian classical music (Raag Malkauns) is associated with modest reductions in systolic and diastolic blood pressure in healthy elderly individuals. Changes in surface temperature and oxygen saturation were minimal and clinically insignificant. While these findings suggest potential for short-term physiological modulation, the absence of a control group and the exploratory design preclude causal conclusions. Larger randomized controlled trials incorporating direct autonomic measures and longer follow-up are warranted to determine sustained clinical relevance.
